# Cell type- and replication stage-specific influenza virus responses in vivo

**DOI:** 10.1371/journal.ppat.1008760

**Published:** 2020-08-13

**Authors:** Elizabeth J. Fay, Stephanie L. Aron, Marissa G. Macchietto, Matthew W. Markman, Katharina Esser-Nobis, Michael Gale, Steven Shen, Ryan A. Langlois

**Affiliations:** 1 Biochemistry, Molecular Biology and Biophysics Graduate Program, University of Minnesota, Minneapolis MN, United States of America; 2 Center for Immunology, University of Minnesota, Minneapolis MN, United States of America; 3 Department of Microbiology and Immunology, University of Minnesota, Minneapolis MN, United States of America; 4 Institute for Health Informatics, University of Minnesota, Minneapolis MN, United States of America; 5 Department of Immunology and Center for Innate Immunity and Immune Disease, University of Washington, Seattle WA, United States of America; Emory University School of Medicine, UNITED STATES

## Abstract

Influenza A viruses (IAVs) remain a significant global health burden. Activation of the innate immune response is important for controlling early virus replication and spread. It is unclear how early IAV replication events contribute to immune detection. Additionally, while many cell types in the lung can be infected, it is not known if all cell types contribute equally to establish the antiviral state in the host. Here, we use single-cycle influenza A viruses (scIAVs) to characterize the early immune response to IAV *in vitro* and *in vivo*. We found that the magnitude of virus replication contributes to antiviral gene expression within infected cells prior to the induction of a global response. We also developed a scIAV that is only capable of undergoing primary transcription, the earliest stage of virus replication. Using this tool, we uncovered replication stage-specific responses *in vitro* and *in vivo*. Using several innate immune receptor knockout cell lines, we identify RIG-I as the predominant antiviral detector of primary virus transcription and amplified replication *in vitro*. Through a Cre-inducible reporter mouse, we used scIAVs expressing Cre-recombinase to characterize cell type-specific responses *in vivo*. Individual cell types upregulate unique sets of antiviral genes in response to both primary virus transcription and amplified replication. We also identified antiviral genes that are only upregulated in response to direct infection. Altogether, these data offer insight into the early mechanisms of antiviral gene activation during influenza A infection.

## Author summary

Influenza A virus (IAV) is a respiratory virus that can infect multiple cell types in the lung. It is not known how individual cell types contribute to the innate immune response. Additionally, it is not known how distinct stages of early IAV replication are detected by infected cells. To address these questions, we use a combination of viruses that can replicate but not spread and viruses that can only undergo the very first steps of replication. These tools allow us to identify and analyze infected cells during the earliest stages of virus replication. We uncover distinct responses to different stages of IAV replication *in vitro* and *in vivo*. Additionally, we identify universal and cell type-specific antiviral responses *in vivo*. Altogether, our data suggest that both replication stage and the cell type infected contribute to antiviral gene activation during IAV infection.

## Introduction

Influenza A virus (IAV) is a seasonal pathogen that causes significant global morbidity and mortality annually. Respiratory epithelial cells are the primary targets of IAV. Infected epithelial cells play a critical role in detecting IAV and activating the interferon (IFN) response. Failure of infected cells to control IAV replication can lead to cell death and spread of the virus to neighboring cells, resulting in significant damage to the lung epithelium and severe disease symptoms. The primary innate immune receptor responsible for detection of IAV infection in epithelial cells is retinoic acid inducible gene-I (RIG-I). Detection through another RIG-I-like receptor (RLR), melanoma differentiation-associated protein 5 (MDA5), has also been shown to contribute *in vivo* but not in *ex vivo* studies [1, 2]. While many epithelial cell types can be infected throughout the course of infection, it is unknown if all infected cell types contribute equally to establish the antiviral state in the host.

IAV has a segmented, negative-sense RNA genome. Each of the eight gene segments is packaged into virions in complex with the heterotrimeric viral RNA-dependent RNA polymerase (RdRp). Upon infection, these viral ribonucleoprotein (vRNP) complexes traffic to the nucleus where the RdRp both transcribes the viral RNA (vRNA) to generate messenger RNA (mRNA) and replicates the vRNA through a positive sense complementary RNA (cRNA) intermediate [3]. While the exact mechanism for how the virus balances between transcription and replication for each gene segment is unknown, replication requires *de novo* polymerase complexes to stabilize the cRNA intermediate [4–7], suggesting that transcription occurs prior to replication. Additionally, amplification of vRNA has been shown to be required for induction of type I IFN, suggesting early IAV infection is poorly detected by the innate immune system [6, 8]. Several groups have described aberrant vRNA products, including defective interfering genomes and mini viral RNAs, as the predominant inducers of innate immune activation through RIG-I [9–11]. When these RNAs are produced during the course of an infection has not been well defined.

Previous methods to assess distinct stages of early virus replication within a cell have used drugs such as actinomycin D or cycloheximide to inhibit transcription or translation [11–13]. These drugs also inhibit host cell processes, limiting the ability to analyze the host response. We therefore used a series of viruses genetically restricted in progressing through different stages of replication. Single-cycle influenza viruses (scIAVs) lacking hemagglutinin protein and unable to spread were used to elucidate mechanisms of innate immune activation during the early stages of IAV infection in mice. We identified unique responses to the magnitude of replication during direct infection *in vivo*, prior to the establishment of tissue-wide antiviral responses. Additionally, we generated a genetically restricted scIAV such that only primary transcription can occur. Entry and primary transcription alone are detected by RIG-I and drive an antiviral response *in vitro*. Using this tool, we uncovered epithelial cell type-specific responses to primary virus transcription and amplified virus replication *in vivo*. Altogether, these data demonstrate that the antiviral response to IAV is sensitive to the stage of replication and varies across cell types.

## Results

### Heterogeneous antiviral response to early influenza infection *in vivo*

**Fig 1:**
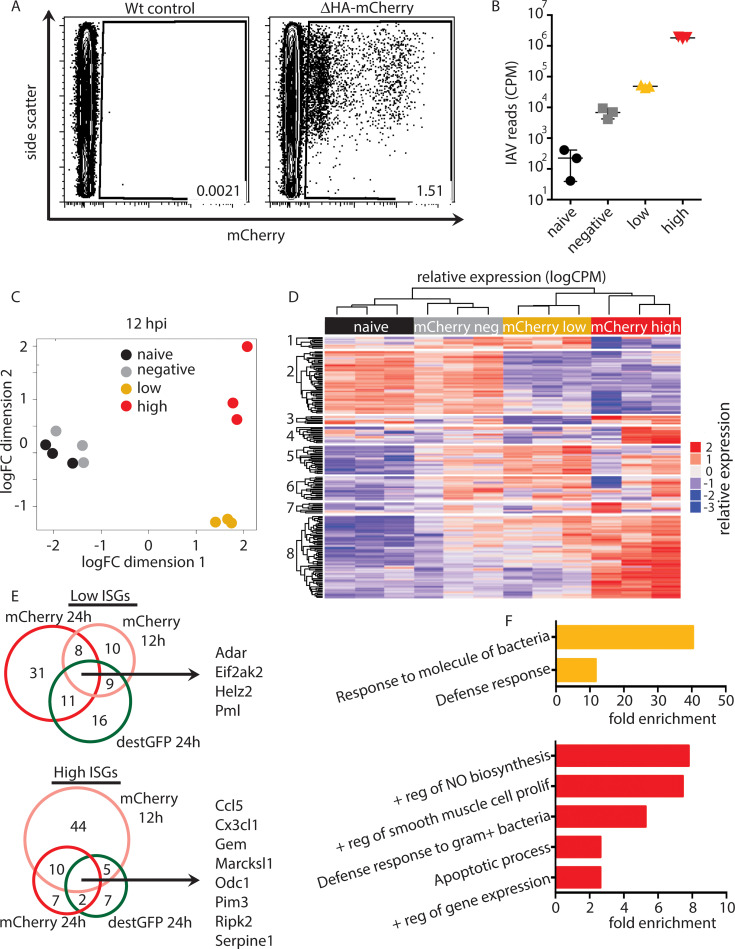
Heterogeneous antiviral response to scIAV infection at 12 hpi. B6 mice were infected with 10^5^ PFU of scIAV-ΔHA-mCherry or 10^3^ PFU PR8. (A) CD45^-^CD31^-^ cells were analyzed for mCherry expression at 12 hpi. Data representative of six independent experiments with n = 3–4 mice per group. mCherry negative, low, and high CD45^-^CD31^-^cells were sorted at 12 hpi for RNA-seq analysis. (B) IAV CPM (C) MDS of naïve and mCherry negative, low, and high cells. (D) Heatmap of 207 ISGs differentially expressed in the indicated populations. Cutoff of false discovery rate (FDR) is ≤ 0.05. (E) Overlapping low ISGs (mCherry 24h cluster 2, GFP cluster 1, and mCherry 12h clusters 1 and 5) and high ISGs (mCherry 24h cluster 4, GFP cluster 4b, and mCherry 12h cluster 8). Only genes induced to ≥10 CPM in at least one sample are shown. (F) Gene ontology analysis (DAVID, biological processes) was performed for genes upregulated in mCherry high, low, and negative cells over naïve (logFC ≥ 1.5, FDR ≤ 0.05). Unique pathways identified in mCherry low (top) mCherry high (bottom) are shown (FDR ≤ 0.05). (B-F) representative of one experiment with n = 3 mice per group.

We have previously used a single-cycle influenza A virus (scIAV) expressing mCherry in place of the coding sequence for the hemagglutinin (HA) segment (ΔHA-mCherry) to uncover replication heterogeneity during the early stages of IAV infection in mice [14]. Other groups have found similar heterogeneity at early timepoints *in vitro* [15–18], as well as heterogeneity in the ability to induce IFN production in infected cells [18–21]. Our previous analyses were unable to distinguish genes induced directly by virus infection from those driven by IFN and inflammation. To address this, we assessed an earlier time point, 12 hours post-infection (hpi), where distinct populations of mCherry high and low epithelial cells were still observed *in vivo* (Fig 1A). To determine if mCherry high and low cells display distinct antiviral signatures, we infected mice with ΔHA-mCherry and sorted mCherry high, low, and negative epithelial cells at 12 hpi for mRNA-seq analysis. Similar to 24 hpi, at 12 hpi reads mapping to the IAV genome were higher in the mCherry high cells than in mCherry low cells, validating the use of mCherry fluorescence as an indicator of scIAV replication at 12 hpi (Fig 1B). Multidimensional scaling (MDS) of host mRNAs revealed significant differences between the mCherry high and low populations (Fig 1C). However, there is no difference between the mCherry negative and naïve populations, suggesting that alterations in host gene expression in mCherry^+^ cells at 12 hpi are driven directly by virus replication, rather than a global inflammatory response. Moreover, mCherry high and low cells display distinct antiviral gene signatures (Fig 1D). While the genes analyzed in this study are designated as interferon-simulated genes (ISGs), IFN-independent upregulation by virus replication of some of these genes has been described [22–24] and we do not distinguish between virus induced and interferon induced. The putative protective ISGs found in the mCherry low cells at 12 hpi overlap with the genes identified using both ΔHA-mCherry and ΔHA-destabilized GFP at 24 hpi identified previously [14] (Fig 1E, top, S1 Table). Among these are genes such as *Eif2ak2* (PKR) which has well-described antiviral activity during IAV infection [25], as well as genes that have not been described to have anti-IAV activity, such as *Helz2*. We also found overlap in the genes upregulated in cells with high levels of virus replication (Fig 1E, bottom, S2 Table), including the chemokine *Ccl5*. Gene ontology (GO) analysis of genes significantly upregulated over naïve in mCherry high and low cells revealed that only high levels of virus replication induce apoptosis pathways at 12 hpi (Fig 1F). Induction of these ISGs may require high levels of replication as a way to tightly regulate a pro-inflammatory and pro-apoptotic response. These data further suggest that pathologic responses may be driven from only a small subset of infected cells. Altogether, these data suggest antiviral gene expression is tuned to the level of IAV replication, and this heterogeneity is established prior to the induction of a global immune response.

### Genetic restriction of IAV to primary transcription

**Fig 2:**
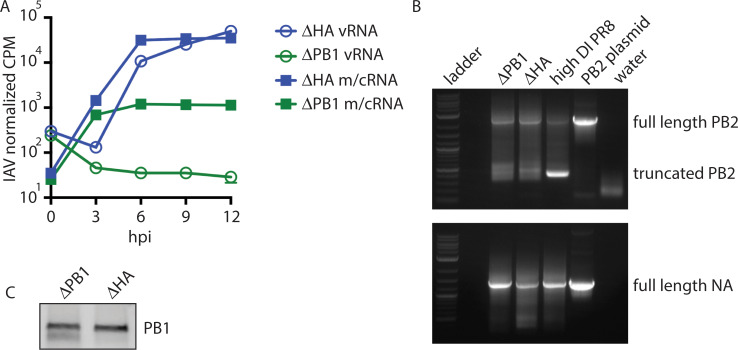
Genetic restriction of IAV to primary transcription. (A) A549 cells were infected with ΔHA-mCherry or ΔPB1-mCherry at MOI = 1 and harvested at the indicated timepoints for RNA-seq. Positive sense (m/cRNA) and negative sense (vRNA) RNA was quantified. Data representative of two independent experiments with n = 3 replicate samples per group. (B) vRNA was extracted from the indicated virus stock and PB2 (top) and NA (bottom) were amplified. Representative of two independent experiments. (C) 1.24x10^5^ PFU of ΔPB1-Cre and ΔHA-Cre virus were analyzed for PB1 protein. Representative of three independent experiments.

We hypothesized that the heterogeneous scIAV replication is due to differential ability of cells to detect and respond to the very early stages of virus replication. We therefore developed a scIAV that is unable to progress from primary transcription to replication. We replaced the coding sequence for polymerase basic 1 (PB1) with the coding sequence for mCherry (ΔPB1-mCherry). This virus is grown in a cell line that expresses PB1 protein. The resulting viruses package complete RdRps but cannot generate new polymerase complexes in infected cells. Therefore, any *de novo* RNA generated in infected cells is being produced only by incoming virus RdRps. We first quantified levels of (+)-sense (m/cRNA) and (-)-sense (vRNA) RNA generated by ΔHA-mCherry and ΔPB1-mCherry at 0, 3, 6, 9, and 12 hpi in A549 cells. We found that while both viruses can produce (+)-sense RNA, scIAV-ΔPB1-mCherry cannot amplify (-)-sense vRNA (Fig 2A), validating ΔPB1-mCherry as a tool to assess the immune response to primary IAV transcription.

We hypothesized that, due to the different RNA species generated, ΔHA and ΔPB1 viruses would induce distinct immune responses. Importantly, ΔHA-mCherry and ΔPB1-mCherry virus stocks contain similar amounts of defective interfering (DI) genomes (Fig 2B), which have been shown to induce RIG-I activation independent of viral protein synthesis [11]. Additionally, ΔHA and ΔPB1 viruses package equivalent PB1 protein (Fig 2C). Therefore, any differences in immune activation are due to differences in the *de novo* RNA generated by the two viruses rather than differences in incoming defective genomes or polymerase complexes.

### Primary transcription is detected by RIG-I *in vitro*

**Fig 3:**
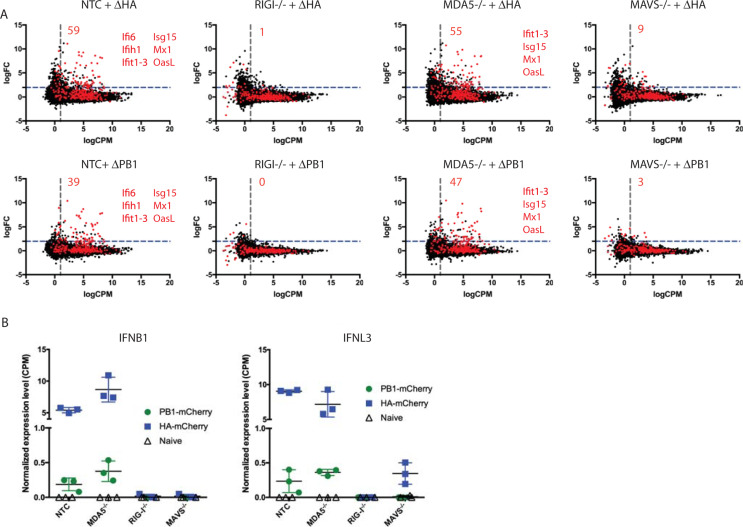
Primary transcription is detected by RIG-I *in vitro*. Indicated A549 cells were infected with ΔHA-mCherry or ΔPB1-mCherry at MOI = 1 and RNA extracted at 12 hpi for mRNA-seq analysis. (A) Total (black) and ISGs (red) differentially expressed genes are shown. The number of genes significantly upregulated (logFC≥2, FDR≤0.05, log CPM≥1.5) over naïve is shown in upper right of plot. The highest upregulated genes (logFC≥5, logCPM≥5) are listed in each plot. (B) Normalized expression level (read CPM) for *IFNB1* and *IFNL3* in naïve and ΔHA-mCherry or ΔPB1-mCherry infected A549 cells. Data representative of one experiment with n = 3 replicate samples per group.

RIG-I, and not MDA5, has been reported to recognize IAV infection in primary mouse fibroblasts [2], but the processes of the viral infection and replication cycle that contribute to this recognition are not known. To understand how ΔPB1-mCherry is being detected by infected cells, we infected RIG-I^-/-^, MDA5^-/-^, MAVS^-/-^, and non-targeted control (NTC) A549 cells [26] with ΔPB1-mCherry or ΔHA-mCherry and harvested cells for mRNA-seq analysis at 12 hpi. Importantly, naïve knockout cells do not display significant differences in overall gene expression compared to naïve NTC cells (S1 Fig). Compared to naïve cells, both viruses robustly induce antiviral gene expression in NTC cells (Fig 3A). The highest upregulated genes (logFC≥5, logCPM≥5) in NTC and MDA5^-/-^ cells are identical in ΔHA and ΔPB1 infections. Additionally, the only gene upregulated in NTC cells that is not upregulated in MDA5^-/-^ cells is *IFIH1* (MDA5). No genes are upregulated to this degree in RIG-I^-/-^ or MAVS^-/-^ during either infection (S3 Table). This suggests that RIG-I is required to detect IAV infection. In both NTC and MDA5^-/-^ cells, ΔHA infection upregulates *IFNB* and *IFNL3* (Fig 3B). ΔPB1 infection also significantly upregulates these IFNs (logFC≥2 over naïve, FDR≤0.05), albeit to a much lower degree. However, only a small percentage of infected cells produce IFN at early timepoints *in vitro* and *in vivo* [18, 19], suggesting that only a few IFN-producing cells are needed to establish the antiviral state. Our data indicate the modest upregulation of IFN in ΔPB1-infected NTC and MDA5^-/-^ cells is sufficient to induce upregulation of ISGs and that the early stages of IAV infection—entry through primary transcription—are sufficient for detection through RIG-I.

### Detection of cells supporting primary transcription *in vivo*

**Fig 4:**
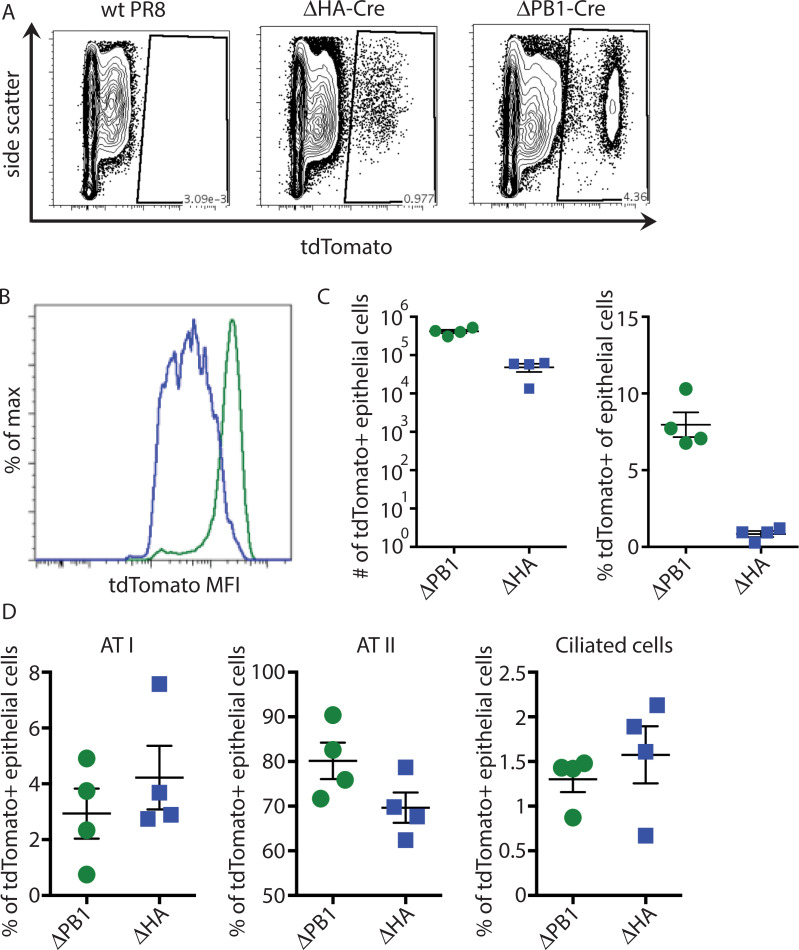
Detection of cells supporting only primary transcription *in vivo*. (A-D) Cre reporter mice were infected with 10^3^ PFU of PR8 or 10^5^ PFU of ΔHA-Cre or ΔPB1-Cre and lungs analyzed by flow cytometry at 24 hpi. (A-B) Epithelial cells (CD45^-^CD31^-^) were analyzed for tdTomato expression. (C) Number and percentage of CD45-CD31- epithelial cells that are tdTomato+. (D) The percentage of infected (tdTomato^+^) ATI, ATII, and ATII cells was quantified. (A-D) representative of 3 independent experiments with n = 3–4 mice.

While we were able to detect virus mRNA by RNA-seq, we were unable to detect mCherry fluorescence in A549 cells infected with ΔPB1-mCherry (S2A Fig). The mCherry gene segment is appropriately packaged, as co-infection with wt IAV to trans-complement PB1 results in mCherry fluorescence. Due to the limited ability to detect ΔPB1-mCherry *in vitro*, we developed additional scIAVs for *in vivo* analysis. These viruses express Cre recombinase (Cre) in place of either HA or PB1 (ΔHA-Cre and ΔPB1-Cre, respectively). Cre-inducible reporter mice have previously been used to identify cells infected with IAV expressing Cre [27–30]. This system allows for the tracking of infected cells via a Cre-inducible host-endogenous fluorophore, tdTomato. Therefore, detection of infected cells is not dependent on high levels of active virus replication. tdTomato^+^ lung epithelial cells can be detected at 24 hpi with either ΔHA-Cre or ΔPB1-Cre (Fig 4A). However, the geometric mean fluorescence intensity (gMFI) of tdTomato is higher in ΔPB1-Cre infected mice (Fig 4B). As tdTomato is a host-endogenous fluorophore, this gMFI difference could reflect differences in the ability of ΔHA and ΔPB1 viruses to induce shut-off of host transcription/translation. Using cell type-specific markers, we identified infected ciliated cells (CD24^hi^ podoplanin^-^), type I alveolar cells (ATI; CD24^-^ podoplanin^+^), and type II alveolar cells (ATII; CD24^-^ podoplanin^-^ MHCII^+^ EpCAM^+^) (S2B Fig for gating strategy). There are overall fewer tdTomato^+^ cells following ΔHA-Cre infection, likely due to more robust cell death from full replication compared to ΔPB1-Cre infection (Fig 4C). However, the proportion of each epithelial cell type within the tdTomato^+^ population was the same in the two infections (Figs 4D, S2C), suggesting these cell types are equally susceptible to infection-induced cell death. All ΔHA-Cre infected cell types show lower tdTomato gMFI compared to ΔPB1-Cre infected cells. Intriguingly, ΔHA-Cre infected ciliated cells show reduced gMFI compared to total ΔHA-Cre tdTomato^+^ cells. Similarly, ΔPB1-Cre infected ATI and ciliated cells show reduced tdTomato gMFI compared to total tdTomato^+^ cells (S2D Fig), suggesting that there are cell type-specific responses to primary scIAV transcription and replication.

### Transcriptional response to viral replication and primary transcription in epithelial cell subsets *in vivo*

We sorted tdTomato^+^ and tdTomato^-^ ATI, ATII, and ciliated cells from ΔHA-Cre and ΔPB1-Cre infected Cre-inducible reporter mice for mRNA sequencing to characterize cell type- and stage-specific responses to scIAV infection. Cells from naïve mice were used as baseline controls. We and others have previously used CD24 as a marker of ciliated cells [14, 31], and we further validated its use by quantifying co-expression of CD24 with the ciliated cell marker acetylated alpha-tubulin (aat) (S3A and S3B Fig). We also validated our gating strategy by quantifying cell type-specific gene expression; we identified cell type-specific expression of both transcription factors and cell surface proteins associated with each cell type (S3C Fig) [32–34]. Expression of innate immune signaling genes in naïve cells could contribute to any differences in the response between cell types. We therefore quantified expression of such genes in each cell type in naïve animals. ATI cells express higher basal *Ddx58* (RIG-I) and *Ifih1* (MDA5) than ATII cells or ciliated cells; other signaling genes—*Irf3*, *Irf7*, and *Mavs*—are not different between the cell types (S3D Fig). Overall, these data confirm enrichment of the indicated epithelial cell types to analyze innate immune responses to scIAVs.

**Fig 5:**
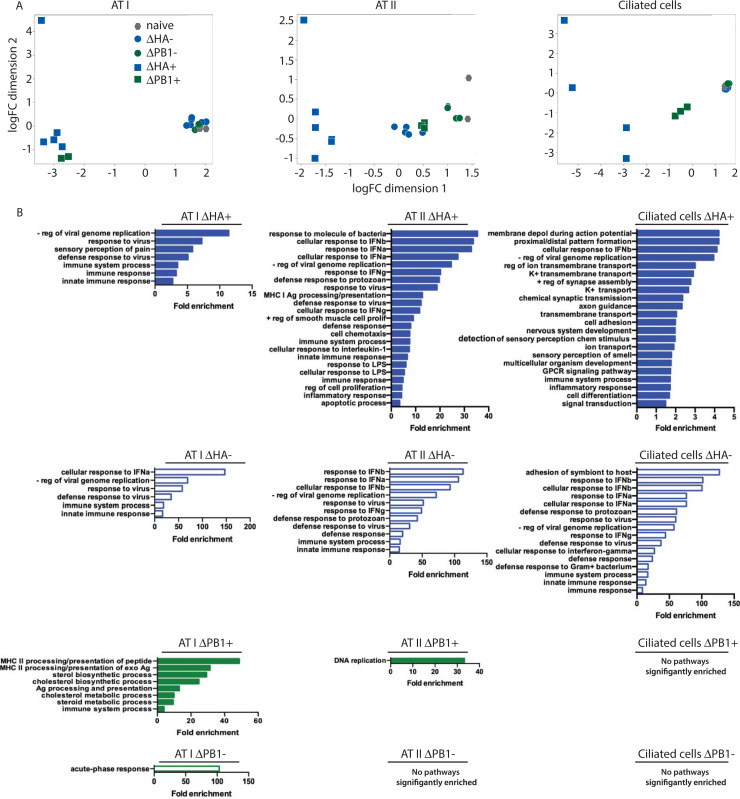
Transcriptional response to viral replication and primary transcription in epithelial cell subsets *in vivo*. Cre reporter mice were infected with 10^5^ pfu ΔHA-Cre or ΔPB1-Cre and tdTomato^+^ and tdTomato^-^ ATI, ATII, and ciliated cells were sorted at 24 hpi for RNAseq analysis. (A) MDS plots of the indicated cell type. ΔHA+: infected, ΔHA-: uninfected, ΔPB1+: infected, ΔPB1-: uninfected. (B) GO analysis (DAVID, biological processes) of significantly upregulated genes (logFC≥1.5, FDR≤0.01, CPM≥10 in at least one sample) for the indicated population of each cell type. Significantly enriched pathways (FDR≤0.05) are shown.

To assess cell type-specific responses to infection, we first looked at global changes in host transcripts. ATI cells respond robustly to direct infection, with similar responses to primary transcription and amplified replication (Fig 5A, left). In contrast, ATII cells have a tiered response to direct infection, and uninfected ATII cells from ΔHA-Cre infected mice also have a large response to global inflammation (Fig 5A, middle). Ciliated cells respond to direct infection, and this response is tiered to different stages of virus replication (Fig 5A, right). Quantification of IAV gene expression—excluding HA and PB1 reads—in each cell type revealed no differences between ΔHA-Cre-infected cells, while ΔPB1-Cre-infected ciliated cells have higher IAV reads than ΔPB1 infected ATI or ATII cells (S4A Fig). ATI, ATII, and ciliated cells all upregulate *Ifnb* in response to ΔHA-Cre infection (S4B Fig). In contrast to the *in vitro* data (Fig 3), ΔPB1-Cre is unable to induce detectable *Ifnb* in the analyzed cell types *in vivo*, which may be due to lower relative MOI. Importantly, these cell types also do not upregulate detectable levels of the IFN-dependent ISG *Mx1* in response to ΔPB1-Cre infection, indicating that interferon is likely not secreted by any cell type (S4C Fig). Both infected and uninfected cells upregulate *Mx1* in response to ΔHA-Cre, suggesting that amplified virus replication is required to induce IFN production *in vivo* and any genes upregulated in ΔPB1-Cre infected cells, including genes designated as ISGs, are likely due to direct infection.

We also performed GO analysis on genes significantly upregulated in each condition over naïve (Fig 5B). As anticipated, antiviral immune pathways were the most significantly upregulated in infected and uninfected cells from ΔHA-infected mice. Compared to other infected cell types, ΔHA^+^ ciliated cells upregulate a more diverse range of genes. Conversely, ΔPB1 infected mice did not have the same degree of immune pathway activation. ΔPB1 infected ATI cells activated expression of pathways involved in metabolism not seen in any other cell type or condition. Altogether, these data suggest different cell types have distinct responses to infection and is impacted by the stage of replication.

### Cell type-specific responses to stages of replication *in vivo*

**Fig 6:**
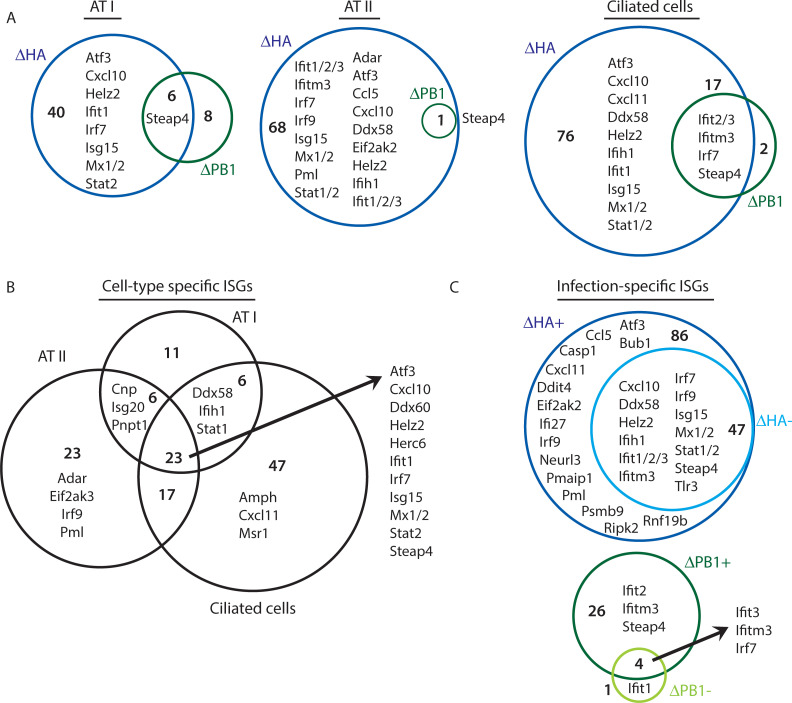
Cell type-specific antiviral responses to stages of virus replication *in vivo*. RNA-seq data from Fig 5 were analyzed for ISG expression (A) Unique and overlapping ISGs significantly upregulated (logFC≥1.5, FDR ≤0.05, CPM≥10 in at least one population) in ΔHA- or ΔPB1-infected tdTomato^+^ cells compared to naïve indicated cell type. (B) Unique and overlapping ISGs significantly upregulated in ΔHA-infected tdTomato+ cells. (C) Infection-specific ISGs were identified by comparing genes in tdTomato^+^ (ΔHA/ΔPB1^+^) and tdTomato^-^ (ΔHA/PB1^-^) cells. Select genes in each population listed. Data representative of one independent experiment with n = 2–6 mice per group.

To further characterize the immune response induced in different epithelial cell types, we analyzed the ISGs upregulated by primary transcription or amplified replication. We identified ISGs that were specific to ΔHA-Cre or ΔPB1-Cre infection, as well as genes upregulated by both infections (Fig 6A, S4 Table). The previously identified putative protective ISGs (Fig 1E) are all upregulated exclusively by ΔHA-Cre infection. This could indicate that induction of a strongly antiviral response is dependent on *de novo* vRNA production and/or upregulation of IFN. We also identified genes that are only significantly upregulated in ΔPB1-infected cells. These genes may be upregulated in response to virus entry, early trafficking of vRNPs, or some other early stage of virus infection. Following detection of virus RNAs, the genes upregulated in response to RLR signaling may dominate the transcriptome, which is why we do not see significant upregulation of these ΔPB1-specific genes in ΔHA infected mice.

We compared ISGs upregulated in ΔHA-Cre infected IAV^+^ cells between cell types to identify cell type-specific responses to infection (Fig 6B, S5 Table). All cell types upregulate genes involved in antigen processing and presentation (*Herc6*, *Psmb8*, *Rnf213*, *Tap1*). Ciliated cells were the only cells to upregulate several genes involved in endocytosis and vesicle transport (*Amph*, *Msr1*). ATI and ATII cells specifically upregulated RNA metabolism-associated genes (*Cnp*, *Isg20*, *Pnpt1*), which may be employed as a way to target and degrade virus RNAs, although this has not been tested. In response to ΔHA-Cre infection, all cell types were able to upregulate the putative protective ISG *Helz2* (Fig 1E, S1 Table). However, only ATII cells upregulated *Adar*, *Eif2ak2*, and *Pml*, which all have known anti IAV activity [25, 35, 36]. While all cell types upregulate *Stat2* and the positive RLR regulator *Ddx60*, only ATII and ciliated cells upregulate *Ddx58* (RIG-I), *Ifih1* (MDA5), and *Stat1*. Intriguingly, while ATI cells express higher basal levels of *Ddx58* and *Ifih1* compared to ATII and ciliated cells, ATI cells induce fewer ISGs than other cell types, suggesting response robustness is at least partially independent of RLR expression levels. Overall, these data suggest that different cell types may employ unique strategies that are compatible with the cell function to make the cell inhospitable to virus replication.

We were also able to compare ISGs upregulated in IAV^+^ or IAV^-^ cells over naïve cells as a way to identify infection-specific ISGs (Fig 6C, S6 Table). Many innate signaling genes are upregulated in both infected and uninfected cells from ΔHA-Cre infected mice (RLRs, *Irf7*, *Tlr3*). Among the infection-specific ISGs upregulated during ΔHA-Cre infection are genes associated with apoptosis (*Ripk2*, *Casp1*, *Ifi27*, *Pmaip1*) and E3 ubiquitin ligases and proteasome genes, some of which are known to be involved in MHC-I antigen processing (*Neurl3*, *Rnf19b*, *Psmb9*). While many known ISGs with anti-IAV activity are upregulated in both IAV^+^ and IAV^-^ cells, *Eif2ak2* is infection-specific. Repair of virus-induced DNA damage is critical for cell survival from IAV infection [37], and several DNA damage response-associated genes (*Bub1*, *Ddit4*, *Pml*) were found to be infection-specific. Overall, the cell type infected, the stage of IAV replication, and direct infection all contribute to the antiviral response of a cell *in vivo*.

## Discussion

The early innate response in infected cells is critical for controlling IAV pathogenesis *in vivo*. Following import into the nucleus, the IAV RdRp first transcribes vRNA to generate mRNA and protein. After *de novo* polymerase complexes are generated, the vRNA is replicated. These two distinct stages of early virus replication are of critical importance in innate immune signaling, as *de novo* vRNA is necessary for induction of IFN-β during IAV infection. IFN-β production within the first hour of IAV infection has been documented in both human and mouse cells [38]. Additionally, entry of vRNPs alone can induce IFN expression [39]. RIG-I is known to detect short (10-300bp) cytosolic RNAs with 5’ triphosphate ends [40]. While aberrant (-)-sense IAV RNA products—DI genomes and mini viral RNAs—are detected by RIG-I, the contribution of (+)-sense IAV RNAs to innate immune detection is unclear. Triphosphate-independent recognition of lariat structures derived from vRNA and cRNA has been described, and both potently upregulate IFN-I [41]. ΔPB1 viruses are able to generate (+)-sense RNA, therefore cRNA-derived structures could be driving the response to ΔPB1 infection.

IFN-independent, IRF-dependent upregulation of some ISGs has been described [22–24]. In these studies, detection of viral RNA products is still required for virus-induced gene expression. We are unable to detect IFN expression following ΔPB1 infection *in vivo*, and the observed induction of antiviral genes could therefore be driven directly through RLR signaling. Membrane perturbations—such as those that occur during virus binding and entry into the host cell—have been shown to be sufficient to induce IRF3-mediated gene activation *in vitro* [42], and this response is independent of RLR signaling [43]. The upregulation of ISGs we see during ΔPB1 infection could therefore be due to entry-associated membrane perturbations rather than detection of RNA species, and these responses would be revealed in RLR signaling deficient cells. As RIG-I knockout and MAVS knockout cells do not significantly upregulate any genes following ΔPB1-mCherry infection, virus entry induces little response *in vitro*. The RIG-I-dependent response to ΔPB1 infection could be through incoming vRNP complexes, independent of polymerase activity. An additional facet of the early innate immune response to viruses is the upregulation of endogenous transposable elements [44] and endogenous retroviruses [45]. As primary transcription is sufficient for induction of antiviral genes, transposable elements and endogenous retroviruses are likely also upregulated and contributing to the cellular response.

Various techniques have been employed to assess distinct stages of IAV infection within a cell. These studies use inhibitors to prevent protein synthesis or transcription, which effectively halt virus replication but also target cellular processes [11–13]. This limits the ability to analyze the immune response, as expression of host genes is compromised. Specific inhibition of virus replication could be achieved by using drugs that target the IAV polymerase specifically, either by targeted drug design or by engineering IAVs to be susceptible to drug control, as described through the use of the small molecule assisted shutoff tag. Addition of this tag to an IAV polymerase gene resulted in an IAV whose replication was susceptible to hepatitis C virus protease inhibitors [46], allowing for specific inhibition of IAV replication. In addition to inhibition of replication, recent studies using single-cell RNA sequencing technologies have uncovered cellular responses to IAVs lacking one or more gene segments, including viruses lacking polymerase segments. Similar to our results using ΔPB1 scIAVs, cells infected with viruses lacking vRNP genes produce less virus mRNA and induce little to no IFN [15]. However, a virus lacking both PB1 and NS1 potently induced IFN [18]. While our data suggest that primary transcription can induce antiviral gene expression in an IFN-independent manner, in the absence of immune antagonism, primary IAV transcription may be sufficient to drive IFN expression. Unfortunately, we do not know the percentage of infected cells that will fail to progress beyond primary transcription during wt IAV infection. While the early detection we observe here is likely critical to rapidly induce IFN, sensing of full replication may drown out other primary transcription-specific responses later in infection.

We and others have identified cell type-specific responses to IAV infection *in vivo* [14, 31]. Unlike previous studies, we are able to compare three different epithelial cell types, the stage of replication (primary virus transcription vs amplified replication), and assess the response of bystander cells. Some of these cell type differences may be explained by an incompatibility with certain ISGs and the function of a given cell type (*e*.*g*. expression of *Eif2ak2* may be incompatible with critical ciliated cell function). The only ISG upregulated by all cell types in during both full replication and primary transcription is *Steap4*. STEAP4—also known as STAMP2—is a metalloreductase that has antiviral activity during hepatitis B virus (HBV) infection. STEAP4 prevents HBV-induced metabolic dysregulation and can antagonize HBV gene expression, thereby protecting cells from HBV [47]. Expression of *Steap4* in scIAV-infected cells could serve a similar function.

Basal levels of signaling genes may contribute to the observed differential antiviral responses between cell types. Surprisingly, while ATI cells express higher levels of *Ddx58* and *Ifih1* (S3D Fig), ciliated cells upregulate the most ISGs, even in uninfected bystander cells. These data suggest that the expression level of innate immune receptors alone does not dictate the robustness of the response. In addition to upregulating more ISGs than other epithelial cell types, the highest upregulated ISG in ΔPB1-Cre-infected ciliated cells is *Ifitm3*. IFITM3 is known to inhibit entry/uncoating of IAV [48, 49]; the potent upregulation of *Ifitm3* during early stages of infection and/or the rapid upregulation of ISGs could explain our previously described protection of ciliated cells during virus spread [14]. The epigenetic landscape of different cell types prior to and during early infection could also contribute to differences in gene expression, as epigenetic differences affect functional outcomes during virus infections [50].

Overall, we have described the use of a genetically restricted scIAV to assess cell type- and virus replication stage-specific host responses to infection. We determined that both primary virus transcription and amplified replication are detected through RIG-I. Additionally, we found that the magnitude of early replication, the stage of replication, and the cell type infected all contribute to the antiviral response *in vivo*. Altogether, these data offer insight into the mechanisms of innate immune activation during influenza infection.

## Methods

### Tissue culture

Human embryonic kidney 293T (293T, ATCC) cells, human lung adenocarcinoma A549 cells, Madin-Darby canine kidney (MDCK) cells, and MDCK cells expressing IAV-WSN HA (WSN-HA MDCK, kind gift from Dr. Adolfo García-Sastre, Mount Sinai) were maintained in Dulbecco’s modified Eagle’s medium (DMEM) with 1% fetal bovine serum (FBS) and 1% penicillin-streptomycin. MDCK cells expressing IAV-PR8 HA (PR8-HA MDCK, kind gift from Dr. Luis Martinez-Sobrido, University of Rochester) were supplemented with 125 μg/mL of hygromycin B.

### Generation of PR8-PB1 MDCK cells

The PR8-PB1 coding sequence was cloned into the NotI-digested pLEX-MCS lentivirus packaging vector (kind gift from Dr. Wade Bresnahan, University of Minnesota) using In-Fusion cloning (Takara). The lentivirus packaging vector and the pMDG and pΔNRF helper plasmids were transfected into 293T cells using the Lipofectamine 3000 transfection reagents (Invitrogen). At 24 and 48 hours post-transfection, supernatant was harvested and filtered through a 0.45 μm PES filter. A GFP lentivirus (GFP-lenti) was generated as a control to determine the approximate titer of the PB1-lenti stock. MDCK cells were transduced with 10-fold serial dilutions of GFP-lenti to determine titer. MDCK cells were transduced with PB1-lenti at an MOI = 0.5. After 48 hours, the cells were diluted to obtain single-cell clones. Positive clones were selected for and maintained in Dulbecco’s modified Eagle’s medium (DMEM) with 1% fetal bovine serum (FBS), 1% penicillin-streptomycin, and 5 μg/mL puromycin. Integration of the lentivirus transgene was verified by western blot (rabbit anti-PB1, PA5-34914, ThermoFisher Scientific) and PCR using genomic DNA and the following primers for PB1: 5’-ACCCATAACGATAGAAAAAACTTGTG-3’ and 5’-CCAGTTGGAGGCAATGAGAAGAAAGC-3’.

### Virus rescue

Viruses were rescued in 293T cells using the IAV-PR8 plasmid-based transfection system in the pDZ vector. scIAV-ΔHA viruses were generated as previously described [14]. To generate scIAV-ΔPB1 viruses, mCherry or Cre recombinase (Cre) was inserted between the 5’ and 3’ packaging signals of PR8 PB1 (100 and 200 bp, respectively). Plasmids were transfected at 500ng/reaction onto 293T cells in Opti-MEM using Lipofectamine 2000 (Invitrogen) and incubated at 37°C. pCAGGs-WSN-PB1 or -WSN-HA were supplemented into each reaction. After 24 hours, PR8-PB1 MDCK or WSN-HA MDCK cells were added to transfected wells in Opti-MEM containing 0.5μg/mL TPCK-trypsin. Reactions were supplemented at 24 and 48 hours after cell overlay with 500μL of Opti-MEM containing 1–2μg/mL TPCK trypsin. Seventy-two hours after cell overlay, the supernatant was harvested, centrifuged to remove cellular debris, and stored at -80°C. Viruses were plaque purified and amplified on either PR8-PB1 or PR8-HA MDCK cells. Viral sequences were confirmed using Sanger sequencing. Virus stocks were tittered via plaque assay. Infections were performed in infection media (PBS with 10% Ca/Mg, 1% pen/strep, 5% BSA) at 37°C on either PR8-PB1 or WSN-HA MDCK cells. After 1hr, infection media was replaced with an agar overlay (2xMEM, 1μg/mL TPCK-trypsin, 1% DEAE-dextran, 5% NaCO_3_, 2% oxoid agar) and cultured for 40-42hrs at 37°C. Plaques were fixed with 4% formaldehyde for 30 minutes prior to removal of the overlay. Blocking and immunostaining were performed at room temperature for 1 hour in 5% milk in PBS. The following antibodies were used in staining: polyclonal anti-IAV PR8/34, 1:5000 (V301-511-552), Peroxidase Rabbit Anti-Chicken IgG, 1:5000 (303-035-003, Jackson Immuno Research). Virus plaques were detected using TruBlue Peroxidase Substrate (50-547-28, Kirkegard & Perry Laboratories).

### Stranded sequencing analysis to identify (+)- and (-)-sense scIAV RNA

Infections were performed in A549 cells in infection media at an MOI of 1. Infections were synchronized at 4°C for 30 minutes then transferred and incubated at 37°C. The zero hour time point was harvested after 30 minutes at 37°C and additional time points were harvested at 3, 6, 9, 12 hours post infection. RNA was extracted using TRIzol. The cDNA libraries were prepared using the Stranded Total RNA v2 PicoMammalian kit (Takara). Samples were sequenced as 150 base pair paired-end reads using NovaSeq (Illumina). The customized influenza A/PR/8/34/(H1N1) mRNA sequence and annotation were used for mapping (available upon request). The forward and reverse virus reads counts were obtained by using FeatureCounts -s parameter. The strand specific reads of individual viral genes were summed as either negative or positive strand reads sample by sample, respectively. To normalize the strand specific viral reads, we first generated the forward or reverse strand reads ratio by using total viral reads as denominator. The ratio of forward or reverse strand reads were then normalized against total mapped reads (relative library size), which were subsequently transformed into counts per million (CPM). All RNA sequencing files are available from the NCBI GEO database (accession number GSE147832).

### Western blot analysis

Viral stocks were lysed and separated by SDS-PAGE (2–15% gel). Protein was transferred to a nitrocellulose membrane at 4°C for 2 hours and blocked with 5% milk in PBS. The membrane was incubated with primary antibodies rabbit anti-PB1 (1:1000, PA5-34914, ThermoFisher Scientific) followed by goat anti-rabbit IgG horseradish peroxidase-conjugated secondary antibody (1:1000, ThermoFisher Scientific). Images were obtained using a Li-Cor Odyssey Fc imaging system.

### Detection of Defective Interfering Particles in scIAV stocks

RNA was extracted from viral stocks, including an A/Puerto Rico/8/34 stock grown in MDCK cells. RNA was extracted using the NucleoSpin Virus Kit (Macheray-Nagel). RNA was reverse transcribed to cDNA using the SuperScript III One-Step RT-PCR with Platinum Taq (Invitrogen). PB2 and NA gene segments were amplified from each sample as well from a pDZ-PR8-PB2 or NP plasmid control using previously described primers: PB2 5’- GTAGATGCAGCGAAAGCAGGTCAATTAT-3’ and 5’-GTAGCAGCAGTAGAAACAAGGTCGTTTT-3’, NA 5’-GTAGATGCAGCGAAAGCAGGGGTTTAAA-3’ and 5’-GTAGCAGCAGTAGAAACAAGGAGTTTTT-3’. The samples were loaded and run on a 1% agarose gel with 0.012% ethidium bromide in Tris-acetate-EDTA buffer. Images were obtained using a GelDoc EZ Imager (BioRad).

### Next-generation mRNA sequencing of A549 KO cells

NTC and knockout A549 cells (kind gift from Michael Gale, Jr., University of Washington) were infected with ΔHA-mCherry or ΔPB1-mCherry at MOI = 1. Cells were harvested at 12 hpi and RNA extracted using the RNeasy PLUS Micro kit (Qiagen). cDNA libraries were prepared using the TakaraBio PicoMammalian kit and sequenced as 150 base pair paired-end reads using NovaSeq (Illumina). The raw sequencing reads were mapped to human genome (GRCh38) using Bowtie aligner (bowtie2 version 2.3.4.1) with local mode, -L 22 and -N 1 parameters[51]. The mapped reads were then assigned to Ensembl gene model (Homo_sapiens.GRCh38.87.gtf) with featureCounts of the Subread software package (version 1.5.1) [52]. The raw reads count tables were merged to generate data matrix and used for subsequent statistical analysis. To obtain significant differentially expressed genes, the experimental groups by design were compared to control group (naïve) and the edgeR (version 3.24.3) of bioconductor package was used for statistical analysis [53, 54]. Raw reads were normalized by using default method in the package prior to generating stats.

### Mice and virus infection

Wild-type C57BL/6J and B6.Cg-*Gt(ROSA)26Sor*^*tm14(CAG-tdTomato)Hze*^/J mice were purchased from The Jackson Laboratory. Mice were infected intranasally (i.n.) with 10^5^ pfu of scIAV unless otherwise indicated. All experiments involving mice were performed as dictated by the University of Minnesota Institutional Animal Care and Use Committee.

### Flow cytometry

Mice were euthanized and lungs were inflated with 2 mL dispase (Corning) and 0.5 mL 1% low melt agarose (Lonza) and allowed to sit covered with an ice pack for two minutes. Lungs were then removed from mouse and transferred to 1 mL dispase and incubated at room temperature for 45 minutes. Next, lungs were incubated in DMEM with DNase I (Sigma-Aldrich) at 95 U/mL and shaken for 10 minutes at room temperature. Lungs were homogenized in GentleMACS dissociator and red blood cells were lysed with ACK buffer. Cells were filtered to obtain a single cell suspension prior to staining. Cells were stained with Ghost Dye Red 780 (Tonbo), followed by the following antibodies against surface markers: CD45 (30-F11), podoplanin (clone 8.1.1), CD24 (M1/69), EpCAM (CD324, clone G8.8), MHCII (I-A/I-E, clone M5/114.15.2) (Biolegend), and CD31 (clone 390, BD Bioscience). Cell counts were obtained using AccuCheck counting beads (Thermofisher Scientific). Data were acquired on a BD LSRFortessa (Becton Dickinson, San Jose, CA).

### Next-generation mRNA sequencing of sorted mouse lung epithelial cells

C57BL/6J mice were infected with ΔHA-mCherry and CD45^-^CD31^-^ mCherry high, low, and negative epithelial cells were FACS sorted at 12 hpi. B6.Cg-*Gt(ROSA)26Sor*^*tm14(CAG-tdTomato)Hze*^/J mice were infected with either ΔHA-Cre or ΔPB1-Cre and the following CD45^-^CD31^-^ populations were FACS sorted at 24 hpi: tdTomato+ ciliated cells (CD24^hi^ podoplanin^-^), type I alveolar cells (CD24^-^ podoplanin^+^), and type II alveolar cells (CD24^-^ podoplanin^-^ MHCII^+^ EpCAM^+^), and tdTomato- ciliated cells and type I and II alveolar cells. RNA was isolated from samples using the RNeasy Plus Micro kit (Qiagen). cDNA libraries were prepared using the TakaraBio PicoMammalian kit and were sequenced as 50 base pair paired-end reads using NovaSeq (Illumina). The raw sequencing reads were mapped to mouse genome (GRCm38) using Bowtie aligner (bowtie2 version 2.3.4.1) with local mode, -L 22 and -N 1 parameters[51]. The mapped reads were then assigned to Ensembl gene model (Mus_musculus.GRCm38.87.gtf) accordingly with featureCounts of the Subread software package (version 1.5.1) [52]. For flu reads mapping and assignment, the customized influenza A/PR/8/34/(H1N1) mRNA sequence and annotation were used. The raw reads count tables were merged to generate data matrix and used for subsequent statistical analysis. To obtain significant differentially expressed genes, the experimental groups by design were compared to control group (naïve) and the edgeR (version 3.24.3) of bioconductor package was used for statistical analysis [53, 54]. Raw reads were normalized to counts per million for each sample by using default method in the package prior to generating stats.

### Statistical analysis

Statistical analyses were completed using GraphPad Prism 7 software. Comparisons between two groups were executed using a two-tailed Student t test. Comparisons between more than two groups were completed using a one-way ANOVA. Additional tests were performed where indicated. Error bars were calculated using SEM.

### Ethics statement

Care and use of the animals was in accordance with the Guide for the Care and Use of Laboratory Animals from the National Research Council and the USDA Animal Care Resource Guide. All experimental protocols involving the use of mice were approved by the Institutional Animal Care and Use Committee at the University of Minnesota (protocol: 1708-35040A. approved 09/14/2017; expires 09/13/2020).

## Supporting information

S1 Fig Basal gene expression between knockout cell lines. Individual gene expression values (CPM) for naïve NTC A549 cells were plotted against naïve RIG-I^-/-^ (left), MDA5^-/-^ (middle), and MAVS^-/-^ (right) A549 cells. R-squared values were calculated using linear regression analysis.
(PDF)

S2 Fig Detection of cells supporting primary transcription *in vivo*. (A) A549 cells were infected with ΔHA-mCherry, ΔPB1-mCherry, or ΔPB1-mCherry and PR8 at MOI = 1 and analyzed at 24 hpi by flow cytometry. Representative of 3 independent experiments with n = 1 sample replicate per group. (B) representative flow plots for identifying indicated cell types. (C) Total numbers of infected ATI, ATII, and ciliated cells following ΔPB1-Cre or ΔHA-Cre infection. (D) tdTomato gMFI of individual cell types compared to total tdTomato^+^ cells in ΔPB1-Cre and ΔHA-Cre infected mice. Data representative of 3 independent experiments with n = 3–4 mice per group. Student’s t test (C) or one-way ANOVA with Dunnett’s multiple comparisons test (D) *p<0.05 **p<0.01 ***p<0.001.
(PDF)

S3 Fig Verification of epithelial cell identity *in vivo*. Mice were infected with 10^5^ PFU ΔHA-mCherry and lungs harvested at 24 hpi for analysis by flow cytometry. (A) The percentage of CD24^hi^ and CD24^-^ cells that are aat^+^ was quantified. Data representative of one independent experiment with n = 3 mice. (B) Representative flow plots from infected (top) and fluorescence minus one control (bottom) mice are shown. Representative of one experiment with n = 1–3 mice per group. (C-D) Lungs from naïve mice were harvested and ATI, ATII, and ciliated cells were FACS sorted for RNAseq analysis. Levels of the indicated cell type-specific (C) and innate immune signaling (D) genes were quantified. Data representative of one independent experiment with n = 3 mice. One-way ANOVA with Tukey’s multiple comparisons test *** p<0.001, ** p<0.01, ns = not significant.
(PDF)

S4 Fig Amplified scIAV replication is required for IFN signaling. (A) IAV CPM in tdTomato^+^ cells infected with the indicated virus. Expression of *Ifnb* (B) and *Mx1* (C) in ATI, ATII, and ciliated cells in the indicated condition. Data representative of one independent experiment with n = 2–6 mice per group.
(PDF)

S1 Table Putative protective ISGs. (DOCX)

S2 Table Putative reserve ISGs. (DOCX)

S3 Table Stage-specific, RLR-dependent ISG expression in vitro. (DOCX)

S4 Table Replication stage- and cell type-specific ISG expression. (DOCX)

S5 Table Cell type-specific ISG expression. (DOCX)

S6 Table Infection-specific ISG expression. (DOCX)
