# Supplementary figures and images for: Cell type- and replication stage-specific influenza virus responses in vivo
Source: PLoS Pathog. 2020 Aug 13;16(8):e1008760. doi: 10.1371/journal.ppat.1008760 (PMC7447048; doi:10.1371/journal.ppat.1008760)

Supplemental Figure 1

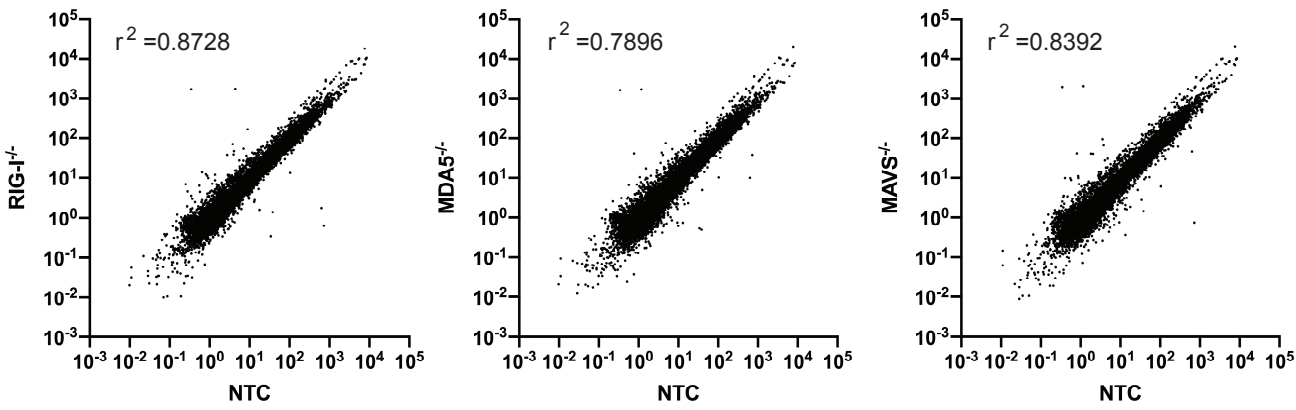

Supplement: S1 Fig — Individual gene expression values (CPM) for naïve NTC A549 cells were plotted against naïve RIG-I-/- (left), MDA5-/- (middle), and MAVS-/- (right) A549 cells. R-squared values were calculated using linear regression analysis. (PDF) [file ppat.1008760.s001.pdf]

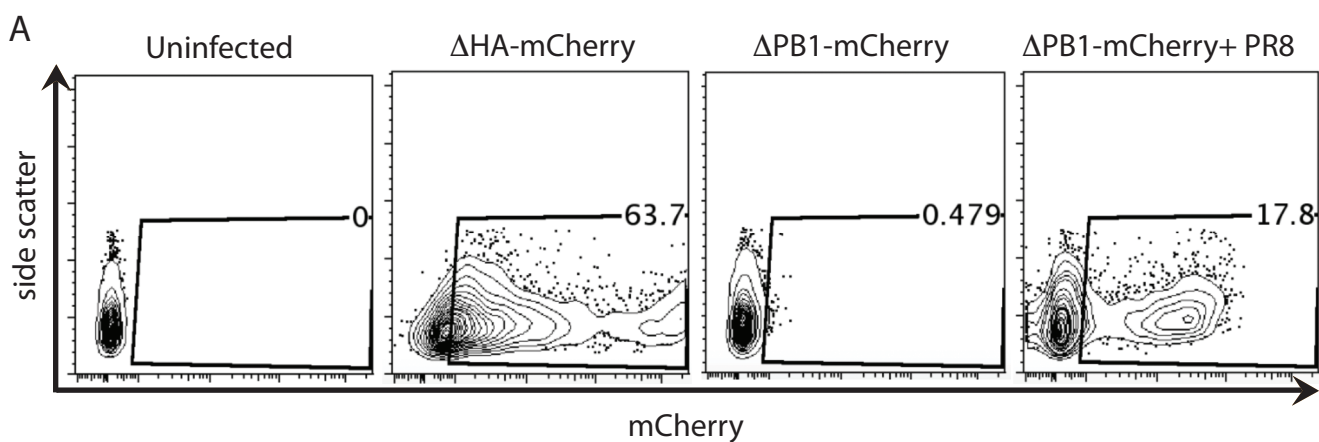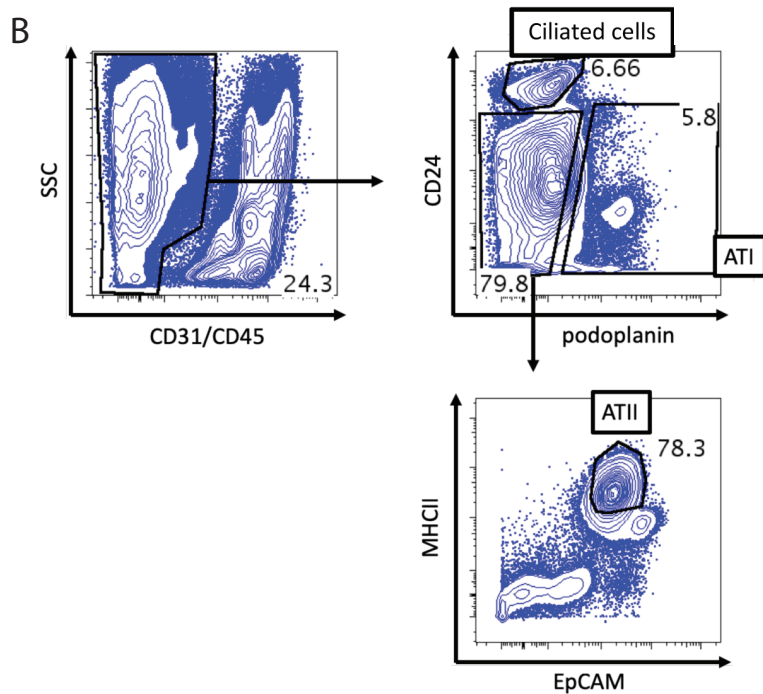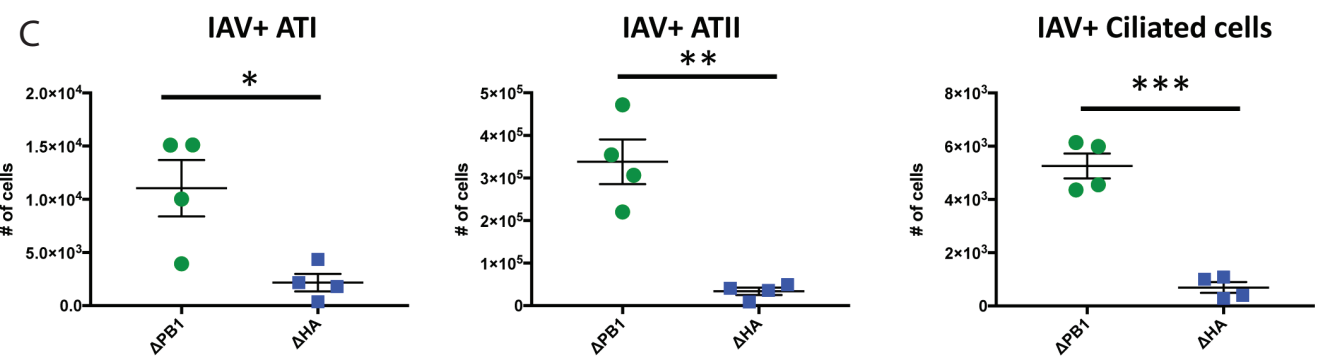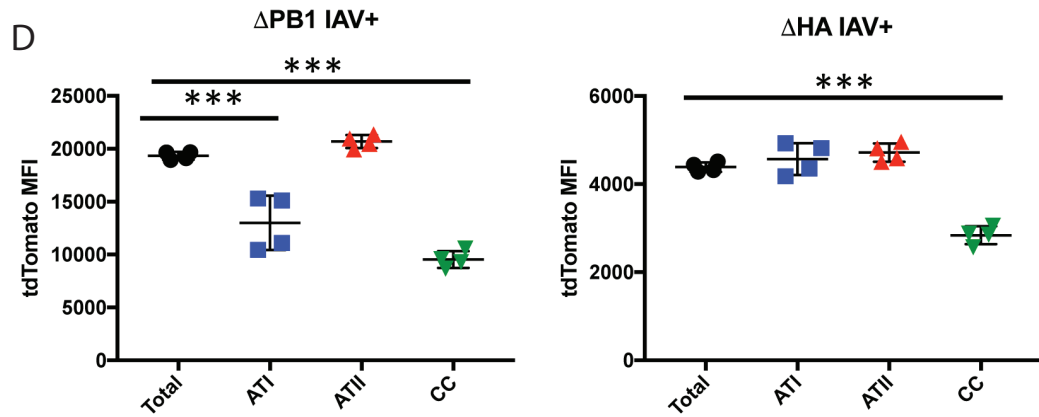

Supplement: S2 Fig — (A) A549 cells were infected with ΔHA-mCherry, ΔPB1-mCherry, or ΔPB1-mCherry and PR8 at MOI = 1 and analyzed at 24 hpi by flow cytometry. Representative of 3 independent experiments with n = 1 sample replicate per group. (B) representative flow plots for identifying indicated cell types. (C) Total numbers of infected ATI, ATII, and ciliated cells following ΔPB1-Cre or ΔHA-Cre infection. (D) tdTomato gMFI of individual cell types compared to total tdTomato+ cells in ΔPB1-Cre and ΔHA-Cre infected mice. Data representative of 3 independent experiments with n = 3–4 mice per group. Student’s t test (C) or one-way ANOVA with Dunnett’s multiple comparisons test (D) *p<0.05 **p<0.01 ***p<0.001. (PDF) [file ppat.1008760.s002.pdf]

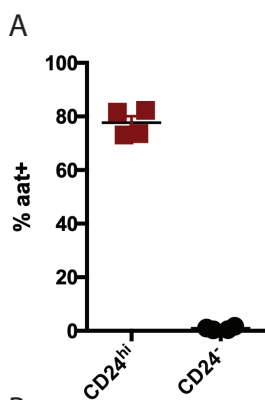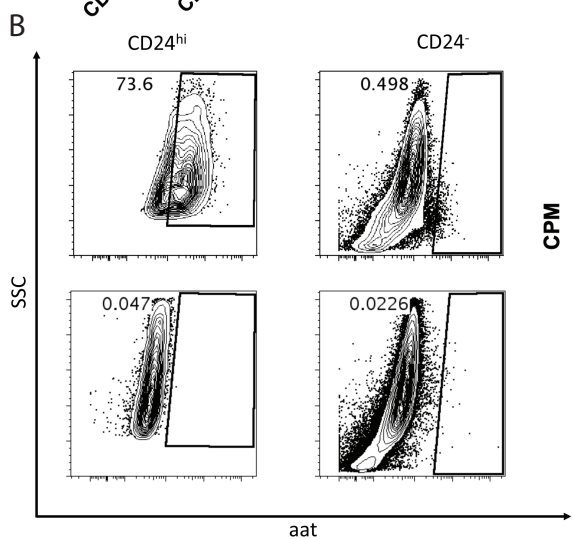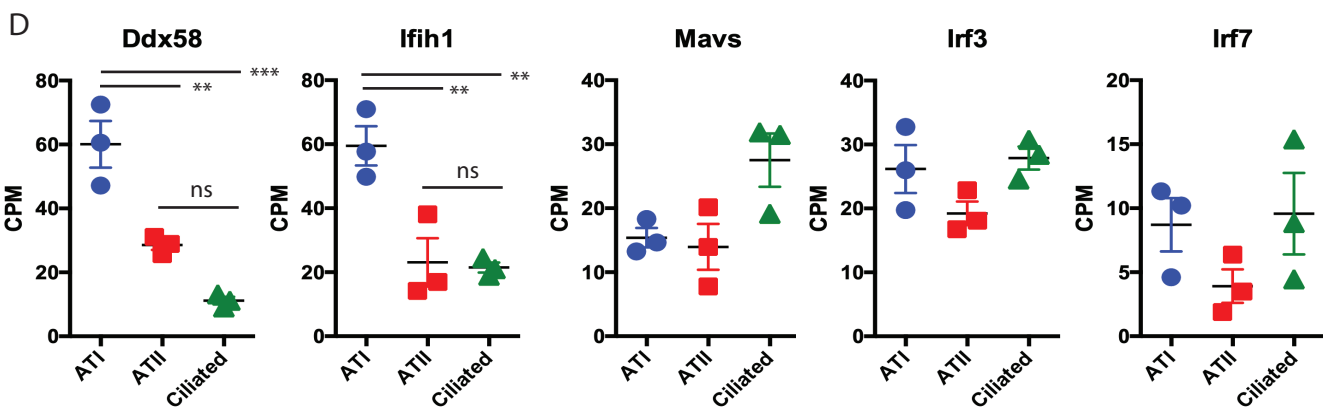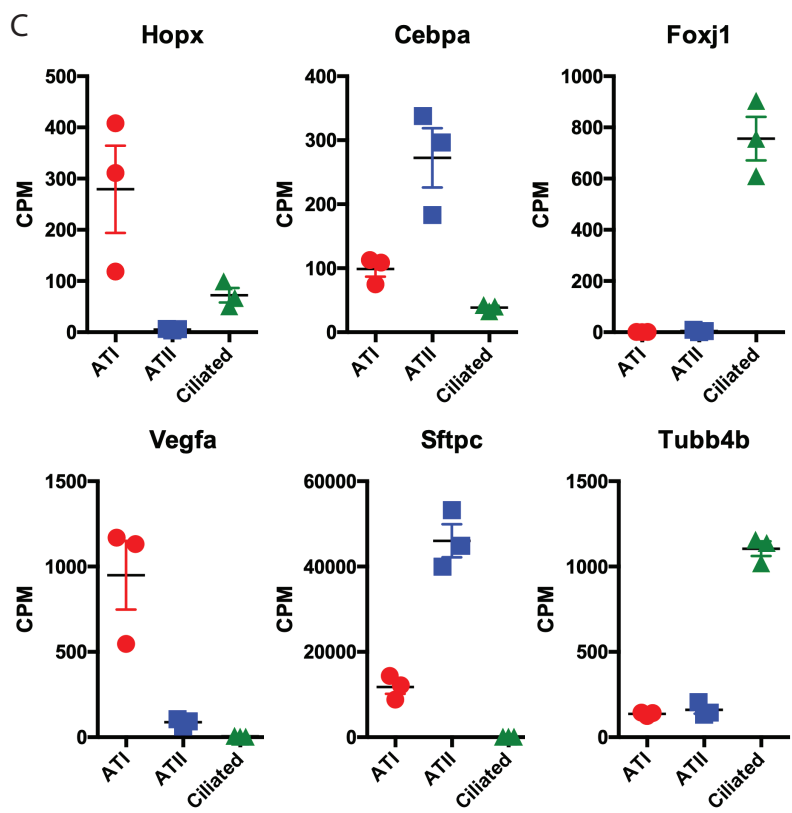

Supplement: S3 Fig — Mice were infected with 105 PFU ΔHA-mCherry and lungs harvested at 24 hpi for analysis by flow cytometry. (A) The percentage of CD24hi and CD24- cells that are aat+ was quantified. Data representative of one independent experiment with n = 3 mice. (B) Representative flow plots from infected (top) and fluorescence minus one control (bottom) mice are shown. Representative of one experiment with n = 1–3 mice per group. (C-D) Lungs from naïve mice were harvested and ATI, ATII, and ciliated cells were FACS sorted for RNAseq analysis. Levels of the indicated cell type-specific (C) and innate immune signaling (D) genes were quantified. Data representative of one independent experiment with n = 3 mice. One-way ANOVA with Tukey’s multiple comparisons test *** p<0.001, ** p<0.01, ns = not significant. (PDF) [file ppat.1008760.s003.pdf]

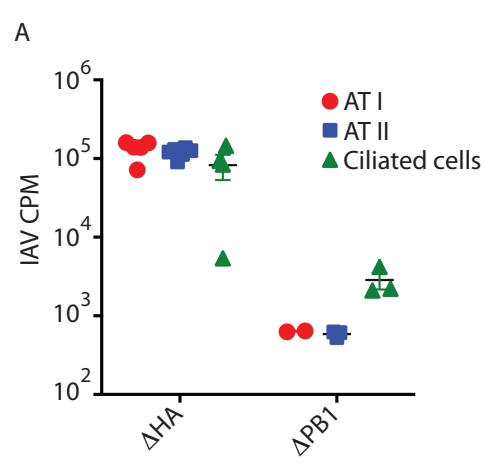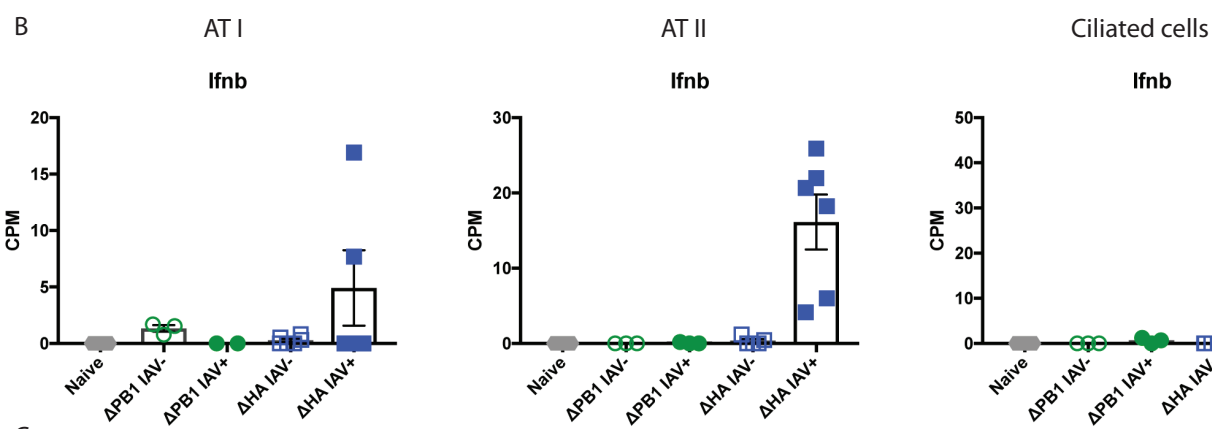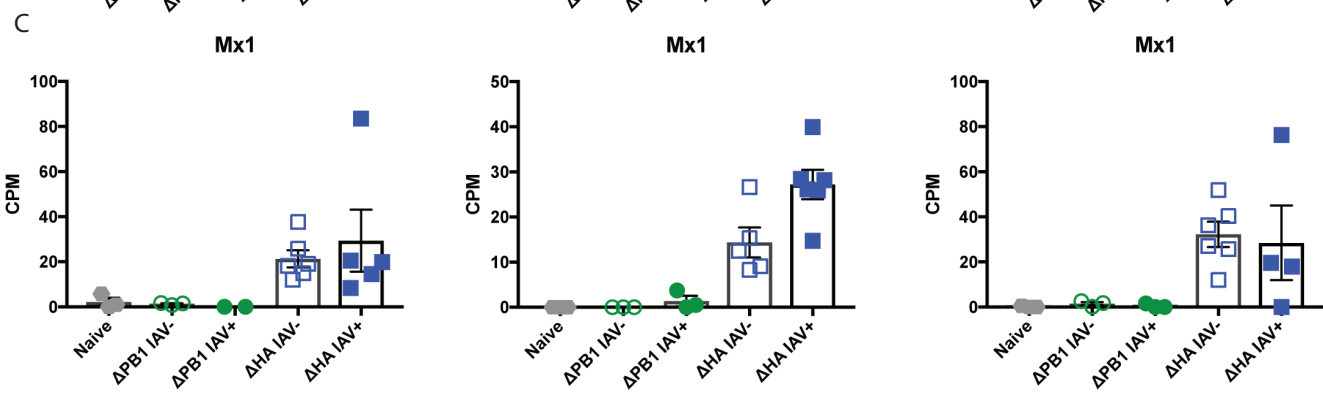

Supplement: S4 Fig — (A) IAV CPM in tdTomato+ cells infected with the indicated virus. Expression of Ifnb (B) and Mx1 (C) in ATI, ATII, and ciliated cells in the indicated condition. Data representative of one independent experiment with n = 2–6 mice per group. (PDF) [file ppat.1008760.s004.pdf]
