# Supplementary material for: Cell type- and replication stage-specific influenza virus responses in vivo
Source: PLoS Pathog. 2020 Aug 13;16(8):e1008760. doi: 10.1371/journal.ppat.1008760 (PMC7447048; doi:10.1371/journal.ppat.1008760)
Supplement: Supplementary file 2 [file ppat.1008760.s005.docx]

ΔHA-mCherry 12 hpi clusters 1 and 5		ΔHA-mCherry 24 hpi cluster 2		ΔHA-destGFP cluster1	
Unc93b1	Lap3	Adar	Lamp3	Adar	Oas3
Adar	Lrg1	Alyref	Lgmn	Birc3	Parp12
Bag1	Ncf1	Ankfy1	Map3k5	Ddx58	Pml
Casp7	Nmi	B4galt5	Max	Ddx60	Pnpt1
Ccnd3	Pml	Bag1	Mov10	Eif2ak2	Prkd2
Cd74	Psmb9	Birc3	N4bp1	Fam46a	Rnf114
Ddx58	Pxk	Ccl2	Ncf1	Helz2	Rnf213
Eif2ak2	Rab27a	Ccnd3	Optn	Herc6	Sp110
Fbxo6	Rnf213	Cd9	Pi4k2b	Ifi27	Spats2l
Glipr2	Serpinb9	Chmp5	Pml	Ifi27l2a	Stat1
Glrx	Stat1	Commd3	Pnpt1	Ifi35	Stat2
Helz2	Stat2	Ehd4	Ptma	Ifit1bl1	Steap4
Ifi27	Tap1	Eif2ak2	Rab27a	Irf2	Tdrd7
Ifi44	Tnfsf10	Eif3l	Rbm25	Irf9	Trim14
Irf9	Uba7	Epas1	Rpl22	Isg20	Trim25
Lamp3		Ext1	Rtcb	Lgmn	Trim5
		Fam46a	Slfn5	Mov10	Trim56
		Fbxo6	Sptlc2	N4bp1	Uba7
		Fndc3b	Sqle	Nmi	Unc93b1
		Gak	Stard5	Oas2	Whamm
		Gca	Stat3		
		Gk	Tdrd7		
		Glipr2	Timp1		
		Glrx	Tmem51		
		Helz2	Trim5		
		Ifi27l2a	Trim56		
		Ifi35	Wars		
