# Supplementary material for: Cell type- and replication stage-specific influenza virus responses in vivo
Source: PLoS Pathog. 2020 Aug 13;16(8):e1008760. doi: 10.1371/journal.ppat.1008760 (PMC7447048; doi:10.1371/journal.ppat.1008760)
Supplement: Supplementary file 3 [file ppat.1008760.s006.docx]

ΔHA-mCherry 12 hpi cluster 8		ΔHA-mCherry 24 hpi cluster 4		ΔHA-destGFP cluster 4b	
Abtb2	Junb	Abtb2	Lgals3	Arg2	Nfil3
Alyref	Lgals9	Atf3	Mafb	Ccl5	Odc1
Arg2	Ly6e	Atp10d	Maff	Cd74	Pim3
Atf3	Mafb	Ccl5	Marcksl1	Cx3cl1	Psmb8
B2m	Maff	Cdkn1a	Mcl1	Ddit4	Ripk2
Bst2	Map3k14	Csrnp1	Nup50	Gem	Rnf24
Ccl5	Marcksl1	Cx3cl1	Odc1	Glipr2	Sat1
Cd274	Max	Ddit4	Pim3	Ifi30	Serpine1
Cdkn1a	Mx1	Fam134b	Ripk2	Lrg1	Slc16a1
Ceacam1	Mx2	Fam46c	Rnf19b	Marcksl1	Slc1a1
Csrnp1	Myd88	Gem	Sat1	Max	Tmem140
Cx3cl1	Nampt	Gpx2	Serpine1		
Cxcl10	Neurl3	Ifngr1	Tnfaip3		
Ddx3x	Nfil3	Junb			
Dtx3l	Nt5c3				
Ehd4	Nup50				
Fkbp5	Odc1				
Gbp2	Pim3				
Gbp3	Psmb8				
Gbp5	Ripk2				
Gch1	Rnf19b				
Gem	Rsad2				
Gk	Rtp4				
Herc6	Samhd1				
Hk2	Serpine1				
Ifih1	Slc16a1				
Ifit1	Steap4				
Ifit1bl1	Tnfaip3				
Ifit2	Trim21				
Ifit3	Ube2l6				
Irf1	Usp18				
Irf7	Xaf1				
Isg15	Zbp1				
