# Supplementary material for: Cell type- and replication stage-specific influenza virus responses in vivo
Source: PLoS Pathog. 2020 Aug 13;16(8):e1008760. doi: 10.1371/journal.ppat.1008760 (PMC7447048; doi:10.1371/journal.ppat.1008760)
Supplement: Supplementary file 4 [file ppat.1008760.s007.docx]

A549 NTC				A549 RIG-I-/-				A549 MDA5-/-				A549 MAVS-/-			
ΔHA-mCherry		ΔPB1-mCherry		ΔHA-mCherry		ΔPB1-mCherry		ΔHA-mCherry		ΔPB1-mCherry		ΔHA-mCherry		ΔPB1-mCherry	
APOL1	IRF7	APOL6	**IFIT3**	ATF3				ADM	IRF1	APOL1	IRF7	ATF3	MX1	IFIT1	MX1
APOL2	**ISG15**	BATF2	IFIT5					APOL1	IRF7	APOL6	**ISG15**	CCL2	OASL		
APOL6	ISG20	C19orf66	IFITM3					APOL2	**ISG15**	BATF2	ISG20	IFIT1	PMAIP1		
ATF3	MAFF	CCL5	IRF7					APOL6	ISG20	C19orf66	**MX1**	IFIT2	TNFAIP3		
BATF2	**MX1**	DDX58	**ISG15**					ATF3	MAFF	CCL5	MX2	IFIT3			
C19orf66	MX2	DDX60	ISG20					BATF2	**MX1**	DDX58	MYD88				
CCL2	MYD88	DTX3L	**MX1**					C19orf66	MX2	DDX60	OAS1				
CCL5	OAS1	GBP1	MX2					CCL2	MYD88	DTX3L	OAS2				
CD274	OAS2	GBP3	OAS2					CCL5	OAS1	FAM46A	OAS3				
DDX58	OAS3	HELZ2	OAS3					CD274	OAS2	GBP1	**OASL**				
DDX60	**OASL**	HERC6	**OASL**					DDX58	**OASL**	HELZ2	PARP12				
DTX3L	PARP12	IFI16	PARP12					DDX60	PARP12	HERC6	PLEKHA4				
FAM46A	PLEKHA4	IFI27	PLEKHA4					DTX3L	PLEKHA4	IFI16	PLSCR1				
GBP1	PLSCR1	IFI35	PLSCR1					FAM46A	PLSCR1	IFI27	PML				
GBP3	PMAIP1	IFI44	RSAD2					GBP1	PMAIP1	IFI35	RSAD2				
HELZ2	PML	IFI44L	SP110					GBP3	PML	IFI44	SP110				
HERC6	PPM1K	**IFI6**	STAT2					HELZ2	PPM1K	IFI44L	STAT1				
IFI16	PSMB9	**IFIH1**	TRIM21					HERC6	RSAD2	IFI6	STAT2				
IFI27	RSAD2	**IFIT1**	USP18					IFI16	SLFN5	IFIH1	TAP1				
IFI35	SAMHD1	**IFIT2**						IFI35	SP110	**IFIT1**	TRIM14				
IFI44	SLFN5							IFI44	STAT2	**IFIT2**	TRIM21				
IFI44L	SP110							IFI44L	TAP1	**IFIT3**	UBE2L6				
**IFI6**	STAT2							IFI6	TNFAIP3	IFIT5	USP18				
**IFIH1**	TAP1							IFIH1	TRIM21	IFITM3					
**IFIT1**	TNFAIP3							**IFIT1**	TRIM38						
**IFIT2**	TRIM21							**IFIT2**	UBE2L6						
**IFIT3**	TRIM38							**IFIT3**	USP18						
IFIT5	UBE2L6							IFIT5							
IFITM3	USP18														
IRF1															
