# Supplementary material for: Cell type- and replication stage-specific influenza virus responses in vivo
Source: PLoS Pathog. 2020 Aug 13;16(8):e1008760. doi: 10.1371/journal.ppat.1008760 (PMC7447048; doi:10.1371/journal.ppat.1008760)
Supplement: Supplementary file 5 [file ppat.1008760.s008.docx]

ATI cells				ATII cells				Ciliated cells			
ΔHA-Cre		ΔPB1-Cre		ΔHA-Cre		ΔPB1-Cre		ΔHA-Cre		ΔPB1-Cre	
Atf3	Maff	Arg2	Nrn1	Abtb2	Mafb	Steap4		Abca9	Junb	Abca9	Ifit1bl1
Ceacam1	Marcksl1	Ceacam1	Pi4k2b	Adar	Maff			Adm	Lamp3	Atp10d	Ifit2
Cebpd	Mov10	Fut4	Psmb8	Atf3	Mx1			Ahnak2	Lgals9	Ccna1	Ifit3
Cnp	Mx1	Gk	Psmb9	B4galt5	Mx2			Amph	Lmo2	Ceacam1	Ifitm3
Cxcl10	Mx2	Lamp3	Rab27a	Bst2	Neurl3			Apol6	Lrg1	Cebpd	Irf7
Ddit4	Nfil3	Lrg1	Sqle	Ccl5	Nt5c3			Arg2	Map3k14	Ddx60	Lamp3
Ddx60	Oas3	Mthfd2l	Steap4	Cd274	Parp12			Atf3	Marcksl1	Fam46c	Lgals9
Dhx58	Psmb8			Cdkn1a	Pfkfb3			Atp10d	Mastl	Gpx2	Slfn5
Fut4	Rgs1			Ceacam1	Pml			Batf2	Mb21d1	Herc6	Steap4
Gch1	Rnf213			Csrnp1	Pnpt1			Bcl3	Msr1	Hk2	
Gem	Rsad2			Cxcl10	Psmb8			Bst2	Mx1		
Gk	Rtp4			Ddx58	Ripk2			Bub1	Mx2		
Gpx2	Stat2			Ddx60	Rnf19b			Casp1	Neurl3		
Helz2	Steap4			Dhx58	Rnf213			Cd38	Nod2		
Herc6	Tap1			Dtx3l	Rsad2			Ceacam1	Oas2		
Ifi44	Tlr3			Eif2ak2	Rtp4			Cebpd	Oas3		
Ifit1	Tnfaip3			Epsti1	Serpine1			Cxcl10	Pim3		
Irf7	Tnfsf10			Gbp2	Slc1a1			Cxcl11	Pmaip1		
Isg15	Trim14			Gbp3	Sp110			Cyp1b1	Psmb8		
Isg20	Usp18			Gbp5	Spats2l			Ddx58	Psmb9		
Lap3	Vmp1			Gch1	Stat1			Ddx60	Rgs1		
Lgals9	Xaf1			Helz2	Stat2			Dtx3l	Rnf213		
Lrg1	Zbp1			Herc6	Steap4			Dusp5	Rsad2		
				Ifi27l2a	Tap1			Epsti1	Samhd1		
				Ifi35	Tlr3			Fam46c	Sat1		
				Ifi44	Tnfaip3			Flt1	Serpinb9		
				Ifih1	Tnfsf10			Gbp2	Serpine1		
				Ifit1	Trafd1			Gbp3	Slc15a3		
				Ifit2	Trim21			Gbp5	Slc16a1		
				Ifit3	Uba7			Gem	Slc25a30		
				Ifitm3	Ube2l6			Gja5	Slfn5		
				Irf7	Usp18			Helz2	Stat1		
				Irf9	Xaf1			Herc6	Stat2		
				Isg15	Zbp1			Hk2	Steap4		
				Lgals9				Hsh2d	Tap1		
								Ifi27	Thbd		
								Ifi44	Tlr3		
								Ifih1	Tlr7		
								Ifit1	Tnfaip3		
								Ifit1bl1	Tnfaip6		
								Ifit2	Tnfsf10		
								Ifit3	Trafd1		
								Ifitm3	Trim21		
								Il15	Ube2l6		
								Il1rn	Vegfc		
								Irf7	Xaf1		
								Isg15			
