# Supplementary material for: Cell type- and replication stage-specific influenza virus responses in vivo
Source: PLoS Pathog. 2020 Aug 13;16(8):e1008760. doi: 10.1371/journal.ppat.1008760 (PMC7447048; doi:10.1371/journal.ppat.1008760)
Supplement: Supplementary file 6 [file ppat.1008760.s009.docx]

ATI cells		ATII cells		Ciliated cells	
Atf3	Maff	Abtb2	Mafb	Abca9	Junb
Ceacam1	Marcksl1	Adar	Maff	Adm	Lamp3
Cebpd	Mov10	Atf3	Mx1	Ahnak2	Lgals9
Cnp	Mx1	B4galt5	Mx2	Amph	Lmo2
Cxcl10	Mx2	Bst2	Neurl3	Apol6	Lrg1
Ddit4	Nfil3	Ccl5	Nt5c3	Arg2	Map3k14
Ddx60	Oas3	Cd274	Parp12	Atf3	Marcksl1
Dhx58	Psmb8	Cdkn1a	Pfkfb3	Atp10d	Mastl
Fut4	Rgs1	Ceacam1	Pml	Batf2	Mb21d1
Gch1	Rnf213	Csrnp1	Pnpt1	Bcl3	Msr1
Gem	Rsad2	Cxcl10	Psmb8	Bst2	Mx1
Gk	Rtp4	Ddx58	Ripk2	Bub1	Mx2
Gpx2	Stat2	Ddx60	Rnf19b	Casp1	Neurl3
Helz2	Steap4	Dhx58	Rnf213	Cd38	Nod2
Herc6	Tap1	Dtx3l	Rsad2	Ceacam1	Oas2
Ifi44	Tlr3	Eif2ak2	Rtp4	Cebpd	Oas3
Ifit1	Tnfaip3	Epsti1	Serpine1	Cxcl10	Pim3
Irf7	Tnfsf10	Gbp2	Slc1a1	Cxcl11	Pmaip1
Isg15	Trim14	Gbp3	Sp110	Cyp1b1	Psmb8
Isg20	Usp18	Gbp5	Spats2l	Ddx58	Psmb9
Lap3	Vmp1	Gch1	Stat1	Ddx60	Rgs1
Lgals9	Xaf1	Helz2	Stat2	Dtx3l	Rnf213
Lrg1	Zbp1	Herc6	Steap4	Dusp5	Rsad2
		Ifi27l2a	Tap1	Epsti1	Samhd1
		Ifi35	Tlr3	Fam46c	Sat1
		Ifi44	Tnfaip3	Flt1	Serpinb9
		Ifih1	Tnfsf10	Gbp2	Serpine1
		Ifit1	Trafd1	Gbp3	Slc15a3
		Ifit2	Trim21	Gbp5	Slc16a1
		Ifit3	Uba7	Gem	Slc25a30
		Ifitm3	Ube2l6	Gja5	Slfn5
		Irf7	Usp18	Helz2	Stat1
		Irf9	Xaf1	Herc6	Stat2
		Isg15	Zbp1	Hk2	Steap4
		Lgals9		Hsh2d	Tap1
				Ifi27	Thbd
				Ifi44	Tlr3
				Ifih1	Tlr7
				Ifit1	Tnfaip3
				Ifit1bl1	Tnfaip6
				Ifit2	Tnfsf10
				Ifit3	Trafd1
				Ifitm3	Trim21
				Il15	Ube2l6
				Il1rn	Vegfc
				Irf7	Xaf1
				Isg15	
