# Supplementary material for: Cell type- and replication stage-specific influenza virus responses in vivo
Source: PLoS Pathog. 2020 Aug 13;16(8):e1008760. doi: 10.1371/journal.ppat.1008760 (PMC7447048; doi:10.1371/journal.ppat.1008760)
Supplement: Supplementary file 7 [file ppat.1008760.s010.docx]

ΔHA-Cre				ΔPB1-Cre			
IAV+		IAV-		IAV+		IAV-	
Abca9	Lamp3	Adar	Lgals9	Arg2	Ifit3	Ddx60	Ifitm3
Abtb2	Lap3	Bst2	Mx1	Abca9	Ifitm3	Ifit1	Irf7
Adar	Lgals9	Cxcl10	Mx2	Atp10d	Irf7	Ifit3	
Adm	Lmo2	Ddx58	Oas2	Ccna1	Lamp3		
Ahnak2	Lrg1	Ddx60	Oas3	Ceacam1	Lgals9		
Amph	Mafb	Dhx58	Parp12	Cebpd	Lrg1		
Apol6	Maff	Dtx3l	Psmb8	Ddx60	Mthfd2l		
Arg2	Map3k14	Epsti1	Rnf213	Fam46c	Nrn1		
Atf3	Marcksl1	Gbp2	Rsad2	Fut4	Pi4k2b		
Atp10d	Mastl	Gbp3	Rtp4	Gk	Psmb8		
B4galt5	Mb21d1	Gbp5	Sp110	Gpx2	Psmb9		
Batf2	Mov10	Helz2	Stat1	Herc6	Rab27a		
Bcl3	Msr1	Herc6	Stat2	Hk2	Slfn5		
Bst2	Mx1	Ifi27l2a	Steap4	Ifit1bl1	Sqle		
Bub1	Mx2	Ifi35	Tap1	Ifit2	Steap4		
Casp1	Neurl3	Ifi44	Tlr3				
Ccl5	Nfil3	Ifih1	Tnfsf10				
Cd274	Nod2	Ifit1	Trafd1				
Cd38	Nt5c3	Ifit1bl1	Uba7				
Cdkn1a	Oas2	Ifit2	Ube2l6				
Ceacam1	Oas3	Ifit3	Usp18				
Cebpd	Parp12	Ifitm3	Xaf1				
Cnp	Pfkfb3	Irf7	Zbp1				
Csrnp1	Pim3	Isg15					
Cxcl10	Pmaip1						
Cxcl11	Pml						
Cyp1b1	Pnpt1						
Ddit4	Psmb8						
Ddx58	Psmb9						
Ddx60	Rgs1						
Dhx58	Ripk2						
Dtx3l	Rnf19b						
Dusp5	Rnf213						
Eif2ak2	Rsad2						
Epsti1	Rtp4						
Fam46c	Samhd1						
Flt1	Sat1						
Fut4	Serpinb9						
Gbp2	Serpine1						
Gbp3	Slc15a3						
Gbp5	Slc16a1						
Gch1	Slc1a1						
Gem	Slc25a30						
Gja5	Slfn5						
Gk	Sp110						
Gpx2	Spats2l						
Helz2	Stat1						
Herc6	Stat2						
Hk2	Steap4						
Hsh2d	Tap1						
Ifi27	Thbd						
Ifi27l2a	Tlr3						
Ifi35	Tlr7						
Ifi44	Tnfaip3						
Ifih1	Tnfaip6						
Ifit1	Tnfsf10						
Ifit1bl1	Trafd1						
Ifit2	Trim14						
Ifit3	Trim21						
Ifitm3	Uba7						
Il15	Ube2l6						
Il1rn	Usp18						
Irf7	Vegfc						
Irf9	Vmp1						
Isg15	Xaf1						
Isg20	Zbp1						
Junb							
